# A Tissue-Targeted Vaccine Protects Against Recurrent Ocular Herpes Infection and Disease, and Alleviates the Associated Cognitive, Behavioral, and Social Deficits in a 'Humanized' HLA Transgenic Mouse Model of UVB-Induced Reactivation

**DOI:** 10.21203/rs.3.rs-11212335/v1

**Published:** 2026-10-21

**Authors:** Sweta Karan, Swayam Prakash, Aziz A. Chentoufi, Khan Intharachalit, Sarah Xue Le Ng, Junseong Hwang, Chhaya Maurya, Lbachir BenMohamed

## Abstract

Recurrent ocular herpetic disease is a leading infectious cause of corneal blindness and is associated with psychological distress, anxiety, and social withdrawal. No ocular herpes vaccine or immunotherapy currently exists. A prime/pull/keep (PPK) T-cell Vaccine, built on HSV-1 epitopes preferentially recognized by CD8 ^+^ /CD4 ^+^ T cells from naturally protected asymptomatic (ASYMP) individuals, would reduce viral reactivation, shedding, recurrent corneal disease, and associated neurobehavioral deficits. Immunodominant HLA-restricted CD8+/CD4 + epitopes from HSV-1 gD/gB, tegument proteins VP11/12/VP13/14, and helicase UL9 were delivered via topical ocular AAV8 vector (prime) to latently infected HLA-A*0201/DRB1 mice, combined with chemokine CXCL11 (pull) and IL-2/IL-15 cytokines to sustain TRM cells (keep), then UVB-exposed to trigger reactivation and recurrent corneal disease. PPK Vaccine conferred robust, near-complete protection against recurrent HSV-1 disease and improved survival after UVB-induced reactivation, unlike its individual components. This protection was associated with site-specific accumulation of HSV-1-specific CD8 ^+^ /CD4 ^+^ T _RM_ cells in the cornea and trigeminal ganglia (TG), but not in the spleen, with peak retention from combined IL-2/IL-15, alongside durable central/effector memory populations. PPK-induced T cells showed enhanced polyfunctionality (cytokine production, cytotoxicity, proliferation), reduced exhaustion markers, and a TCF-1 + self-renewing phenotype. PPK also elicited robust, Th1-biased HSV-1-specific IgG responses complementing mucosal immunity, and restored spatial memory, reduced depression-like behavior, and reversed social withdrawal in mice with recurrent ocular herpes. PPK Vaccine induces potent, durable antiviral T _RM_ cells at primary sites of HSV-1 latency and reactivation, protecting against recurrent ocular herpetic disease and alleviating associated neurobehavioral deficits, supporting its translational potential in humans.

